# Domestication Reduces Plant Immune Receptor Gene Repertoires Across Lineages

**DOI:** 10.1093/gbe/evaf147

**Published:** 2025-07-22

**Authors:** Noah Bourne, Nathanael Walker-Hale, Luke Dunning, Guillaume Chomicki

**Affiliations:** Ecology and Evolutionary Biology, School of Biosciences, University of Sheffield, Sheffield S10 2TN, UK; Department of Bioscience, Durham University, Durham DH1 3LE, UK; Ecology and Evolutionary Biology, School of Biosciences, University of Sheffield, Sheffield S10 2TN, UK; Department of Bioscience, Durham University, Durham DH1 3LE, UK

**Keywords:** plant immunity, evolutionary genomics, cost of resistance, gene loss, crop evolution, relaxed selection

## Abstract

Plant domestication is sometimes associated with a reduction in the diversity of immune receptor genes, critical for pathogen recognition and defense. Yet, the extent and evolutionary forces driving this pattern remain unclear. Using a comparative genomics framework, we analyzed the immune receptor gene repertoires of 15 domesticated crop species and their wild relatives, representing nine plant families. We examined both cell surface pattern recognition receptors and intracellular nucleotide-binding leucine-rich repeat receptors. Our results show that five crops—grapes, mandarins, rice, barley, and yellow sarson—exhibited significantly reduced immune receptor gene repertoires compared to their wild counterparts; however, the overall rate of immune receptor gene loss reflected the background rate of gene loss. Despite this, there is a positive association between domestication duration and immune receptor gene loss. Together, these results suggest that domestication imposes a subtle, cumulative pressure, consistent with relaxed selection rather than a strong cost-of-resistance effect. This study provides insights into how domestication impacts plant immunity, with implications for future crop breeding strategies to enhance disease resistance.

Significance Plant domestication has dramatically changed the evolutionary trajectory of crop species. The negative consequences of domestication can include a reduction in genetic diversity and reduced plant immunity with some evidence inferring resistance comes at a high cost. The plant immune receptor gene (IRG) repertoire is vital for pathogen recognition and defense; however, domestication-related impacts on plant IRG repertoires are contrasting and often focused on a single crop. This study compares the IRG repertoires of diverse crop plants and their respective wild relatives within a comparative genomics framework. The results show some crops do harbor reduced IRG repertoires compared to their wild relatives along with a convergent association between the time since domestication and a reduction in the IRG repertoires of crop plants. Furthermore, IRG loss is consistent with background levels of gene loss, suggesting weak selection against the maintenance of IRG repertoires and a low “cost of resistance” effect.

## Introduction

Plant domestication is characterized by an evolutionary process in which humans—as domesticators—ultimately create an environment to actively manage plant survival and reproduction. This often leads to a series of evolutionary phenotypic changes that include desirable traits and is commonly referred to as the “domestication syndrome” (Hammer 1984; Purugganan 2022). In plants, the domestication syndrome encompasses desirable traits such as larger, sweeter fruits, stems, or tubers, and the loss of unpleasant-tasting compounds (e.g. bitterness) (Darwin 1868; Chomicki et al. 2020), which are further shaped by both natural and artificial selection (Vaughan et al. 2007; Spengler 2020). In addition to directly selected traits, domestication drives a wide range of other evolutionary changes. For example, in cereals like wheat, barley, and rice, domestication suppressed lateral branches or tillers, favoring reduced branching and synchronized tiller maturation (Doust 2007; Fuller et al. 2010). Another important aspect involves the loss of potentially useful pathways (for wild populations) during domestication, such as reduced carotenoid compounds in legume seeds (Fernández-Marín et al. 2014), or the loss of genes those involved in disease resistance, particularly those associated with generalized pests (Gaillard et al. 2017).

The loss of innate immunity genes in crops compared to their wild relatives has been report in an increasing number of crops, including maize (Wang et al. 2018), soybean (Bayer et al. 2021), *japonica* rice (Yu et al. 2008), foxtail millet (Inoue et al. 2015), apple (Whitehead and Poveda 2019), and watermelon (Renner et al. 2021). Despite this recurrence, the factors mediating this loss of innate immune genes are unclear (Hajjar and Hodgkin 2007). The extent to which it affects crop immune gene repertoires, the proximate mechanisms involving such loss, and the ultimate factors driving these changes remain unclear. Selective pressure favoring the loss of innate immunity could stem from three main factors: First, domesticators placing domesticates in arenas with reduced pathogen load (due to early managing practices), which could have led to relaxed selection on immune genes (Karasov et al. 2014); second, the loss of genetic diversity, which is shaped by multiple factors, including the severity of the domestication bottleneck, ancestral introgression, the time since domestication, and the crop's life history (Meyer and Purugganan 2013); and third, the cost of resistance, where resistance is metabolically costly and may be harder to maintain if there are tradeoffs with other important crop traits such as biomass, which may constrain IRG repertoire evolution (Bergelson and Purrington 1996; Tian et al. 2003). Research into IRG loss in crops has traditionally been concentrated on one or a few genes/regions to identify novel resistance loci to specific crop diseases (Hajjar and Hodgkin 2007; Wang et al. 2021). Recent advances in sequencing technologies and comparative genomic approaches have now allowed for the characterization of entire repertoires (Van de Weyer et al. 2019; Shang et al. 2022; Tang et al. 2022; Long et al. 2024; Su et al. 2024).

The genomic repertoire underlying immune responses in plants can be broadly characterized into two classes of immune receptor genes (IRGs) that recognize various pathogen compounds. Pathogen recognition receptors (PRRs) are cell surface proteins that recognize pathogen-associated molecular patterns (PAMPs) and elicit PAMP-triggered immunity. However, this response can be inhibited by various pathogen effector proteins. To combat this, plants deploy an intracellular group of nucleotide-binding leucine-rich repeat receptor (NLR) proteins that recognize these effectors and elicit effector-triggered immunity (Jones et al. 2024).

Although several studies have compared IRG repertoires between crops and their wild relatives, they have commonly focused on one half of the IRG repertoire, usually NLRs (Shang et al. 2022; Tang et al. 2022), or just a portion of the genome (Mizuno et al. 2020), and typically on a single crop. Investigating the entire repertoire is essential because immune responses can require crosstalk between PRR- and NLR-induced pathways (Hatsugai et al. 2017; Ngou et al. 2021; Yuan et al. 2021; Feehan et al. 2023; Dodds et al. 2024), and there is strong evidence that these genes coevolve in plants (Ngou et al. 2022). Additionally, inconsistent genome assembly quality, gene model annotation, functional annotation, and IRG classification methods across studies hinder cross-species comparisons. Despite the importance of IRGs in plant–pathogen interactions, it remains unclear how and to what extent domestication has altered the IRG repertoires of crops compared to their wild relatives across the plant tree of life. To address this knowledge gap, we employ a robust comparative genomic framework to systematically analyze the complete IRG repertoires of 15 domesticated crop species and their wild counterparts, spanning nine plant families. Specifically, we aim to address the following research questions: How does the diversity and composition of IRG repertoires differ between domesticated crops and their wild relatives? Are these differences repertoire wide? What factors are associated with the loss of IRGs? Our results provide new insights into the impact of domestication on plant immunity, with implications for improving crop resistance through breeding strategies.

## Results

### Multiple Crops Harbor Significantly Fewer IRGs Compared to Their Wild Relatives

**Fig. 1.**
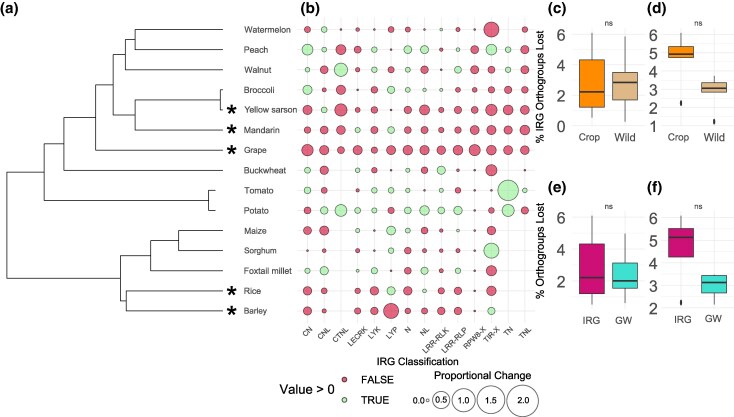
a) Phylogenetic tree of crop species used in the study (see Materials and Methods). b) Proportional changes in each IRG classification between crops and their respective wild relatives. Significantly differing repertoires are marked by *. Proportional increases per IRG classification are marked by green circles and proportional decreases in red circles. c to f) Box plots linking the median to the interquartile range, with whiskers extending to the last points within 1.5 times the interquartile range showing the percentage of IRG orthogroup loss between c) all crops and wild relatives and d) crops with significantly reduced repertoires and their wild relatives. e) All crop IRG orthogroups compared to their own genome-wide (GW) orthogroups and f) crops with significantly reduced repertoires IRG orthogroups compared to their own genome-wide orthogroups. Box plots showing no significant differences are labeled with ns.

We used genome assemblies from 15 crop species representing 9 families across the plant phylogeny (Fig. 1a; Table 1) and reannotated their genomes using a consistent approach to generate counts of loci in 14 IRG classifications (see Materials and Methods) that were compared between crops and their respective wild relatives (Fig. 1b). No significant differences were found in the PRR repertoire, and only two crops showed significant reduction in NLR repertoire (*Vitis vinifera* subsp. *vinifera* [Vitaceae] and *Citrus reticulata* [Rutaceae], one-sample Wilcoxon signed-rank test, both *V* = 45, *P* = 0.029) (Table S4). When considering the entire IRG repertoires, there were significantly fewer IRGs in 5 crop species covering 4 plant families including grapes (*V. vinifera*, Vitaceae, one-sample Wilcoxon signed-rank test *V* = 105, *P* = 0.0018), mandarins (*C. reticulata*, Rutaceae, one-sample Wilcoxon signed-rank test *V* = 97.5, *P* = 0.026), rice (*Oryza sativa*, Poaceae, one-sample *t*-test *t* = 2.92, *P* = 0.046), barley (*Hordeum vulgare*, Poaceae, one-sample *t*-test *t* = 3.23, *P* = 0.0302), yellow sarson (*Brassica rapa* var. *yellow sarson*, Brassicaceae, one-sample Wilcoxon signed-rank test *V* = 88.5, *P* = 0.0222) (Fig. 1b), and none with significantly more IRGs.

**Table 1:** Genome references and BUSCO completeness for plants used in the study

Family	Common name	Latin name	BUSCO assembly (%)	Helixer BUSCO (%)	Wild relative	BUSCO assembly (%)	Helixer BUSCO (%)	Outgroup	BUSCO assembly (%)	Helixer BUSCO (%)
Brassicaceae	Broccoli	*Brassica oleracea* var*. italica*	99.50	99.10	*Brassica oleracea*	99.50	99.30	*Brassica tournefortii*	99.20	98.50
Brassicaceae	Yellow sarson	*Brassica rapa* var*. yellow sarson (Z1)*	99.30	99						
Cucurbitaceae	Watermelon	*Citrullus lanatus* subsp*. vulgaris*	99	95.90	*Citrullus lanatus* subsp*. cordophanus*	98.90	95.10	*Citrullus mucosospermus*	98.90	95.70
Juglandaceae	Black Walnut	*Juglans nigra*	98.70	95.60	*Juglans cinerea*	99	96.90	*Juglans californica*	98.90	97.10
Poaceae	Sorghum	*Sorghum bicolor*	98.30	97.80	*Sorghum bicolor* subsp*. Verticilliflorum*	98.10	97.60	*Coix aquatica*	97.80	97.40
Poaceae	Foxtail millet	*Setaria italica*	98.40	98.30	*Setaria viridis*	98.40	98.80	*Panicum hallii*	98.20	98.30
Poaceae	Rice	*Oryza sativa*	98.70	99	*Oryza rufipogon*	98.50	98.20	*Oryza barthii*	98.70	98.70
Poaceae	Maize	*Zea mays* subsp*. mays* (B73)	98	96.60	*Zea mays* subsp*. Parviglumis*	98.00	96.70	*Zea mays* subsp*. Mexicana*	98.20	96.80
Poaceae	Barley	*Hordeum vulgare* subsp*. vulgare*	98.40	97.40	*Hordeum vulgare* subsp*. Spontaneum*	97.40	96.20	*Hordeum marinum*	98.30	97.80
Polygonaceae	Buckwheat	*Fagopyrum esculentum*	97	95.70	*Fagopyrum homotropicum*	96.90	96.20	*Fallopia multiflora*	97.30	97.60
Rosaceae	Peach	*Prunus persica*	98.10	96.80	*Prunus davidiana*	98.80	98.50	*Prunus mira*	97.90	96.70
Rutaceae	Mandarin	*Citrus reticulata*	98.40	98.40	*Citrus linwuensis*	98	98.10	*Citrus ichangensis*	98.10	98.20
Solanaceae	Tomato	*Solanum lycopersicum*	98.50	97.90	*Solanum galapagense*	98.40	97.50	*Solanum pimpinellifolium*	98.30	97.90
Solanaceae	Potato	*Solanum tuberosum*	96.70	95.60	*Solanum candolleanum*	98.50	96.30	*Solanum buesii*	97.90	98.40
Vitaceae	Grapevine	*Vitis Vinifera* subsp*. vinifera*	98.30	96.20	*Vitis vinifera* subsp*. Sylvestris*	98.80	96.70	*Vitis labrusca*	98.40	95.30

### IRG Loss Reflects the Background Rate of Gene Loss

To test if rates of IRG loss could be explained by an IRG-specific signal instead of genome-wide rates of loss, we calculated rates of gene loss, utilizing synteny-constrained orthogroups (see Materials and Methods), for each crop and wild relative species in relation to their respective outgroup. This was used to calculate (i) the proportion of IRG gene loss in comparison to the proportion of genome-wide gene loss and (ii) the proportion of IRG gene loss in crops compared to their respective wild relatives. When considering all crops and wild relatives in the study, there is no obvious discernible or statistical difference in the rates of gene loss in either category (Fig. 1c and e; Table S6). Interestingly, when considering the five crop species with significantly reduced IRG repertoires, there is a mean increase of 1.867 percentage points (IRG mean loss = 4.665%, SD = 1.453%; genome-wide mean gene loss = 2.797%, SD = 0.661%) in the proportion of IRG gene loss in crops in relation to their own genome-wide levels (paired *t*-test *t* = 2.77, *df* = 4, *P* = 0.053) and a mean increase of 1.521 percentage points (mean crop IRG loss = 4.665%, SD = 1.453%; mean wild IRG loss = 3.144%, SD = 0.404%) to the IRG gene loss in their respective wild relatives (two-sample *t*-test *t* = 2.25, *df* = 4.61, *P* = 0.078) although neither result is statistically significant (Fig. 1d and f).

### Time Since Domestication is Positively Correlated with IRG Loss

**Fig. 2.**
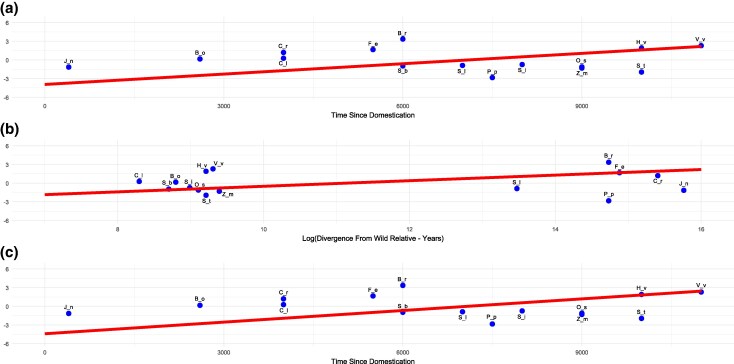
Results from bivariate a, b) and multivariate c) PGLS analysis showing each explanatory variable and their relationship to the percentage difference in IRG loss between crops and their respective wild relatives. The scientific and common species names of the crop species used in the study along with their abbreviations above each data point are as follows: B_o = broccoli/*Brassica oleracea* var. *italica*, B_r = yellow sarson/*Brassica rapa var yellow sarson*, C_l = watermelon/*Citrullus lanatus* subsp. *vulgaris*, C_r = mandarin/*Citrus reticulata*, F_e = buckwheat/*Fagopyrum esculentum*, H_v = barley/*Hordeum vulgare* subsp. *vulgare*, J_*n* = black walnut/*Juglans nigra*, S_b = sorghum/*Sorghum bicolor*, S_i = foxtail millet/*Setaria italica*, O_s = rice/*Oryza sativa*, Z_m = maize/*Zea mays* subsp. *mays*, P_p = peach/*Prunus persica*, S_l = tomato/*Solanum lycopersicum*, V_v = grape/*Vitis vinifera* subsp. *vinifera*, and S_t = potato/*Solanum tuberosum*.

To understand the disparity in IRG loss between crops during domestication, we used a phylogenetic generalized least squares (PGLS) model that tested several explanatory variables against the difference in proportional loss of IRGs in crops versus their respective wild relatives. Explanatory variables were selected based on relevant factors that can be associated with selective pressures toward reduced innate immunity or are a confounding factor. These included biological traits (life history and size of IRG repertoire), possible confounding genome dynamics (assembly genome size and percentage of genes duplicated in the genome), evolutionary history (divergence time from wild relative used in the study and time since domestication), and agricultural factors (minimum crop cycle and the world area harvested). The bivariate (variables tested independently) PGLS models revealed significant positive correlations for two explanatory variables: divergence from the wild relative (Fig. 2b; *t* = 4.66, *P* = 0.0004) and time since domestication (*t* = 3.33, *P* = 0.0054) (Fig. 2a). When analyzing all explanatory variables using a multivariate PGLS model, accounting for their combined effects, time since domestication retained its significant positive correlation (Fig. 2i; *t* = 3.98, *P* = 0.0072). Therefore, only time since domestication is a significant predictor of IRG loss in our results.

## Discussion

The study's key finding is that domestication is contributing to IRG loss in crop plants across diverse lineages and those with significantly reduced IRG repertoires are characterized by a slight (albeit nonsignificant) increase in IRG loss. This subtle convergent increase in IRG loss suggests relaxed selection for the maintenance of IRG repertoire likely due to functional redundancy induced by domestication and the agricultural environment (Ellers et al. 2012; Karasov et al. 2014). Furthermore, since many crops remain unaffected, it suggests that repertoire-wide selection against IRGs is weak, thereby contradicting the “cost of resistance” hypothesis. The PGLS analyses offer further insight, by revealing the significant positive association between the duration of domestication and IRG loss. This suggests two key points: first, domestication contributes to IRG loss in crops, and second, it is an ongoing process with crops under the longest periods of domestication seemingly most affected. The magnitude of the relationship is small when holding all other variables consistent (estimated regression coefficients in multivariate PGLS = 0.00062, *P* = 0.0072) suggesting that even if the “cost of resistance” is a driver, it has a minimal repertoire-wide effect on IRG loss (Fig. 2a and i; Table S7). Our PGLS results also confirm the expected correlation between IRG gene loss and divergence time from wild relatives, reflecting accumulation of genetic differences over evolutionary time.

Reductions in the NLR repertoire of crop plants are supported by recent pangenome studies in rice (Shang et al. 2022), grape (Long et al. 2024), and pan-transcriptome in barley (Ma et al. 2019). While our results follow the same trends as those in the pan-genome studies, we recognize the inherent limitations of using a single accession to represent a species. IRG repertoires can be highly variable both within populations and among very closely related species (Van de Weyer et al. 2019; Tang et al. 2022) so taxon selection will have a notable impact on the results. Despite this, our results both within and across triplicates infer that IRG loss is shaped by relatively weak domestication-related selection, which helps explain why many crop IRG repertoires appear largely unaffected. Additionally, it provides scope for alternative evolutionary explanations behind repertoire-wide IRG expansions. A recent potato pangenome (Tang et al. 2022) showed significant increases across the NLR repertoire. The authors show evidence that clonal propagation could have selectively expanded the NLR repertoire in response to new tuber-borne diseases; however, when including the PRR repertoire in this study, the expansions were found to be nonsignificant. A recent apple pangenome also reports increases across the entire repertoire where the complex history of hybridization and introgression during domestication could be helping to maintain and even expand repertoires (Su et al. 2024). It is also important to acknowledge that not all annotated IRGs will necessarily be functional and while the number of IRGs contributes to disease resistance, other factors, such as the pairing and clustering of IRGs, also play a significant role in shaping immune function (Wersch and Li 2019). As more pan-genomic data of both crops and their wild relatives become available, it will allow for much greater resolution on the evolutionary trajectory of crop IRG repertoires.

It is reasonable to speculate that crop IRG loss is likely influenced by both common and unique domestication-related factors, including diverse domestication pathways (Fuller et al. 2023), locations, pest exposure (Stewart et al. 2025), and physiological and life history changes. For instance, clonal propagation in formerly sexual plants like grapes limits the potential for rescued IRG diversity from outcrossing (Long et al. 2024). Changing rice from a perennial wild plant to annual cultivar in turn halves its generation time, increasing the pace and intensity of domestication-related selection (Kovach et al. 2007). Finally, more common selective pressures induced by domestication bottlenecks, large monocultures, heavy pesticide use, and breeding for increased yield/biomass can all potentially contribute to IRG loss. Remarkably, despite the nonuniform history of crop domestication, a notable trend persists with the positive correlation between IRG loss and time since domestication.

This study enhances our understanding of how domestication impacts the IRG repertoires of crop plants. These findings provide critical insights into identifying crops with the highest levels of IRG loss and the risk of IRG loss for newly domesticated crops and underscore the value of crop wild relatives as IRG reservoirs for enhancing crop breeding efforts (Hajjar and Hodgkin 2007).

## Materials and Methods

### Taxa Selection

To study the impact of domestication on crop IRG repertoires, we focused on five main standards to select the crops: (i) genome quality, (ii) assembly completeness, (iii) consistent gene model annotation, (iv) functional annotation of the entire IRG repertoire, and (v) wide phylogenetic breadth. This involved collating a genome dataset of crop plants, and two of their closest available wild relatives, the more distant one being used as outgroup species (Table S1). The majority of the dataset was diploid (14 triplicates including crop plants, wild relatives, and outgroups), and only one triplicate (potatoes) contained all tetraploids (although haploid assemblies were used, 1*n* = 1*x*). The main aim was to alleviate the impact of polyploidy and to avoid comparisons between plants with different ploidy levels. Plant genomes were then selected from the published literature based on genome quality using only accessions with long-read sequencing or, preferably, chromosome-level assemblies. Crop progenitors were chosen where possible, but when the progenitor was not known or did not have a high-quality genome, the closest related wild relative was chosen based on the most recent phylogenies for the respective crop. Outgroups (i.e. second closest relative of the crop) for each crop wild relative pair were then selected based on the same genome quality and phylogenetic criteria (Table 1).

### Annotation of Gene Models and Quality Control

To ensure consistency in gene model prediction, all downloaded genomes were reannotated with the deep learning based annotation program Helixer v0.3.2 using the land_plant dataset (Holst et al. 2023). BUSCO v5.6.1 (Simão et al. 2015) was then employed to ensure the subsequent proteomes achieved ≥95% complete score (embryophyta_odb10 dataset). Those below this threshold were discarded.

### IRG Functional Annotation

IRGs were annotated by passing the Helixer proteomes through the Drago3 API pipeline using default parameters, which is part of PRGdb 4.0 (Calle García et al. 2021). The raw output was then filtered to only include putative IRG classifications based on domain structures outlined in previous studies (Van de Weyer et al. 2019; Calle García et al. 2021). Domain abbreviations of NLRs are as follows: C = CC/coiled coil, N = NBS/nucleotide-binding site, L = LRR/leucine-rich repeat, TIR = Toll-interleukin-1 receptor, and RPW8 = resistance to powdery mildew. Classifications for PRRs are as follows: LRR-RLP = receptor-like protein (containing LRR and transmembrane domain), LRR-RLK = receptor-like kinase (containing LRR, transmembrane and kinase domain), LYP = lysin motif receptor-like proteins (containing LysM, transmembrane domain), LYK = lysin motif receptor-like kinase (containing LysM, transmembrane and kinase domain), and LECRK = lectin motif receptor-like kinase (containing Lectin, transmembrane and kinase domain). An additional classification CTNL was also used for NBS-LRR proteins that contained both an upstream C and TIR domain. Although LYP and LYK class proteins were generally well predicted, some were required to be manually assigned. As long as a protein had at least one LYSM domain and transmembrane domain predicted from Drago3, it was considered an LYP, and if an additional kinase domain was present, it was considered an LYK. TIR-X proteins were assigned based on the presence of a TIR domain and any additional domain that was not an NBS domain. As Drago3 does not predict RPW8 domains, Interproscan (Blum et al. 2020) was used to identify them, and they were either added to or reassigned in the Drago3 output as RPW8-X.

### Calculating the Difference in IRG Repertoire Between Crops and Their Respective Wild Relative

All statistical tests were performed in R version 4.2.2 (R Core team 2022). To identify significant differences in IRG repertoires, we first normalized the differences in each IRG classification between crops and their respective wild relatives (difference in IRG classification/total sum of differences). We then tested for data normality using a Shapiro–Wilk test. If normality was confirmed (*P* > 0.05), a one-sample *t*-test was applied, otherwise, a one-sample Wilcoxon signed-rank test was used. This allowed us to test if the mean (for one-sample *t*-test) or median (for one-sample Wilcoxon signed-rank test) normalized difference in IRG repertoires was significantly different from 0. The subsequent *P*-values were then corrected for multiple testing using the Benjamini–Hochberg procedure (Benjamini and Hochberg 1995).

### Orthogroup Assignment for IRG and Genome-Wide Gene Loss Calculations

OrthoFinder v 2.5.5 (Emms and Kelly 2019) was first run separately on the monocot and eudicot plants in the study. The OrthoFinder results along with bed and FASTA files of the Helixer annotated proteomes were then used as input for the R package GENESPACE (Lovell et al. 2022), which relies on MCScanX (Wang et al. 2024) to infer synteny and then rerun OrthoFinder within syntenic blocks to assign syntenic orthogroups. Plants were then run in triplicates within this framework (crop wild relative outgroup), and the resulting pan-gene output was parsed by looking at the presence and absence of genes per species in each orthogroup where an outgroup gene was used as the syntenic reference. If a gene was present in the wild relative and outgroup but not in the crop, it was considered a gene loss for the crop; if a gene was present in the crop and outgroup but not in the wild relative, it was considered a gene loss for the wild relative. Outgroup reference genes that were annotated as IRGs were then filtered and gene loss rates were calculated as a proportion of the entire repertoire, i.e. the number of outgroup IRG reference orthogroups. This was then contrasted with genome-wide rates of gene loss as a proportion of genome-wide orthogroups. Significant differences between the mean proportion of IRG gene loss in comparison to genome-wide levels and between crops and their respective wild relatives were calculated via two-sample *t*-tests (paired in the case of IRG loss to genome-wide gene loss comparisons within species) following a Shapiro–Wilk test for normality.

### PGLS

A PGLS model was chosen to explore relationships between IRG loss and explanatory variables as it accounts for shared ancestry between species. The analysis was performed in the R package ape v5.8-1 (Paradis and Schliep 2019) and nlme v3.1-168 (Pinheiro et al. 2025) using a phylogenetic tree of the crop species pruned from Zuntini et al. (2024). If a species was not present in the tree, then the closest available relative within the genus was used as a proxy. References for the explanatory variables data for example the “time since domestication” of each crop can be found in Table S9. An initial linear model was also produced to calculate variance inflation factors using the “*vif*” function (part of the car v3.1-3 R package; Fox and Weisberg 2019), to identify explanatory variables, which heavily correlated with one another, all variables scored <5.5. Bivariate models were first run and then multivariate models where all variables were run simultaneously were performed, using Wald *t*-tests as reporting statistics.

## Supplementary Material

evaf147_Supplementary_Data

## Data Availability

No new sequencing data were generated. References for all Genomes used in the study can be found in Table S1.
